# Solid Lipid Nanoparticles Improve the Diclofenac Availability in Vitreous after Intraocular Injection

**DOI:** 10.1155/2016/1368481

**Published:** 2016-10-10

**Authors:** Mojtaba Abrishami, Majid Abrishami, Asma Mahmoudi, Navid Mosallaei, Mohammad Vakili Ahrari Roodi, Bizhan Malaekeh-Nikouei

**Affiliations:** ^1^Retina Research Center, School of Medicine, Mashhad University of Medical Sciences, Mashhad, Iran; ^2^Eye Research Center, Farabi Eye Hospital, Tehran University of Medical Sciences, Tehran, Iran; ^3^School of Pharmacy, Mashhad University of Medical Sciences, Mashhad, Iran; ^4^Nanotechnology Research Center, School of Pharmacy, Mashhad University of Medical Sciences, Mashhad, Iran

## Abstract

*Purpose*. In order to improve the drug availability after intravitreal administration, solid lipid nanoparticles (SLNs) containing diclofenac were prepared.* Methods*. In this experimental study, 18 albino rabbits were included. In right and left eyes of all rabbits, SLNs containing diclofenac and commercial form of diclofenac (0.3 mg drug) were intravitreally injected, respectively. One, four, twelve, twenty-four, and forty-eight hours after injection, vitreous and aqueous humor samples were obtained in all cases. Then, the concentration of diclofenac sodium was evaluated in all samples.* Results*. Size of nanoparticles was around 170 nm after preparation. Drug concentration in eyes injected with SLNs was significantly higher than left eyes injected with commercial formulation up to 4 hours after intravitreal injection (*p* < 0.05). Diclofenac was quantified in samples up to 48 hours after intraocular injection. Four hours after intravitreal injection, the concentration of diclofenac in vitreous and aqueous humor of eyes receiving SLNs was, respectively, 2.5 and 6.5 times higher than eyes injected with commercial form of drug. * Conclusions*. Here, we demonstrate the potential of SLNs as a carrier of diclofenac for intraocular injection in order to prevent the systemic effects of the drug, increase the injection intervals, and improve the patient compliance.

## 1. Introduction

Diclofenac sodium is an analgesic and anti-inflammatory drug administrable through various dosage forms such as oral, topical, parenteral, suppositories, and eye drops. Recently, diclofenac is used as an intravitreal injection in order to maintain a high intravitreal dose of the drug in naive diabetic macular edema [1], refractory uveitic cystoid macular edema [2], and uveitic cystoid macular edema [3]. Because of restricted half-life of drug after intravitreal administration, achievement of novel drug delivery systems containing this drug for intraocular injection could be a big help in the prevention of local and systemic effects of the drug. Also any increase in the injection intervals improves the patient compliance.

Solid lipid nanoparticles (SLNs) are made of solid lipids with size ranging from 50 to 1000 nm [4]. These nanoparticles have high loading capacity for lipophilic molecules. Chemical protection of labile drug against degradation, high physical stability, easy scaling of the production, and sustained drug release profile are some of the advantages of these colloidal carrier systems [5, 6].

Solid lipid nanoparticles as carrier of diclofenac sodium were used in the study of Attama et al. [7]. These nanoparticles were prepared in order to increase the permeation of ketorolac and diclofenac sodium through cornea. The results of permeation studies showed that the amount of permeated drug was higher at higher drug loading. Efficacy and pharmacokinetics of ketorolac and diclofenac after intraocular injection of the commercial formulation were evaluated in the study of Barañano et al. [8]. The potent anti-inflammatory effect of these drugs after intraocular injection was reported in this study. Also, good penetration of ketorolac and diclofenac into the retina/choroid and rapid clearance of them by 48 h after intravitreal injection were demonstrated.

Intraocular injections have been developed as a method of drug delivery in the treatment of diseases of the retina and macula. Recently, intraocular injection of diclofenac has been considered in these cases. Some studies have shown that this drug is safe for injection into the eye, and fewer complications have reported after intraocular injections compared to corticosteroids such as triamcinolone [3, 9, 10]. The aim of the present study was to prepare SLNs containing diclofenac in order to increase drug residence time after intraocular injection. So, these nanoparticles were prepared and characterized and the kinetics of drug after intraocular injection was evaluated.

## 2. Materials and Methods

### 2.1. Materials

Compritol 888 ATO and Precirol 5 ATO were kindly offered by Gattefosse (France). Hydrogenated soy PC (H-SPC) was purchased from Lipoid (Germany). Poloxamer 188 was provided by Sigma (Germany). Diclofenac sodium injection was from Exir Pharmaceutical Company (Borujerd, Iran), and diclofenac sodium powder was kindly offered by Sobhan Pharmaceutical Company (Rasht, Iran).

### 2.2. Preparation of SLNs Containing Diclofenac

In the first step, diclofenac base (diclofenac acid) was prepared by acidification of an aqueous solution of diclofenac sodium and extraction by chloroform [11]. SLNs containing diclofenac were produced using microemulsion method [12]. Lipid phase was composed of Compritol : Precirol : H-SPC (3 : 5.5 : 1). Diclofenac was added to lipid phase. This phase was melted in 90°C and homogenized using high-speed homogenizer (Ultra-Turrax, IKA, Germany) (20000 rpm, 10 min). Then, isothermal aqueous phase containing 5% w/v Poloxamer 188 as nonionic polymeric stabilizer was added to melted lipid phase. The resulted preemulsion was kept in 25°C for 2 hr and then in 4°C for 24 hr. The preemulsion was heated to 40°C and ultrasonicated in 5 and 10 cycles of 1 min on −1 min off using probe sonicator (MSE, UK).

### 2.3. Characterization of SLNs Containing Diclofenac

The mean size, polydispersity index (PDI), and zeta potential of the prepared SLNs were measured by Zetasizer Nano ZS (Malvern, Worcestershire, UK) after suitable dilution of samples. Size and zeta potential were measured on the basis of Dynamic Light Scattering and Laser Doppler Velocimetry, respectively.

HPLC was used for quantitation of diclofenac base or diclofenac sodium in samples. Calibration curve was plotted in concentration range of 0.02–50 *μ*g/mL. The HPLC condition was as below:

 column (4.6 mm × 250 mm reverse phase stainless steel C18 column), mobile phase (Acetonitrile : Acetic Acid, 60 : 40), flow rate (1 mL/min), and measurement wavelength (270 nm) (retention time of drug in the mentioned condition was 8 min).

To determine the encapsulation efficacy (EE) of drug, desired amount of lyophilized SLNs was weighed and dissolved in chloroform : methanol (1 : 1 v/v) to produce concentration of 0.1 mg/mL if EE was 100%. Twenty *μ*L of clear solution was injected to HPLC, and the real concentration of diclofenac in sample was detected using calibration curve. EE was determined using following formula:

(1) EE%=actual diclofenac concentration in sampleinput diclofenac concentration×100.

### 2.4. Animal Studies

New Zealand albino rabbits weighing 1.8 to 2.0 kg were used in the study. Rabbits were sedated with 60 mg/kg of intramuscular ketamine hydrochloride and 6 mg/mL of intramuscular xylazine hydrochloride. Eyes were anesthetized with topical administration of 1% tetracaine ophthalmic drop. Before the injection, povidone iodine 5% was applied on the surface of the eye for 2 minutes. After washing with balanced salt solution, the eyes of each rabbit received an intravitreal injection of 0.3 mg diclofenac, 2.5 mm behind the limbus in the superotemporal quadrant using a 30-gauge needle. The left eyes were injected with diclofenac sodium (commercial formulation) injection, and right eyes received SLNs containing diclofenac. Eyes were repeatedly monitored for signs of inflammation. Rabbits (3 in each group) were sacrificed at 1, 4, 12, 24, and 48 hours after the intravitreal injection by CO_2_ inhalation. Three animals were used as control (no treatment).

In both eyes, aqueous humor was aspirated with a 30-gauge needle via an anterior limbal paracentesis. Then, the whole of the vitreous was aspirated through incision 4 mm from the limbus. The aqueous humor and vitreous were then immediately frozen at −80°C. Before analysis, the frozen aqueous humor and vitreous were defrosted overnight at 4°C. The next day, samples were centrifuged at 14000 rpm for 30 minutes. Diclofenac sodium concentrations were measured in whole vitreous and aqueous fluid by HPLC. The animal protocol was approved by the Ethical Committee of Mashhad University of Medical Sciences (Mashhad, Iran).

### 2.5. Statistical Analysis

All statistical analysis was done by one-way ANOVA test. Mean ± SD was compared using InStat software (version 3.1). The *p* value less than 0.05 considered to be statistically significant.

## 3. Results

Table 1 shows the characteristics of SLNs containing diclofenac. Size of nanoparticles was around 170 nm after preparation. Zeta potential of prepared nanoparticles was neutral and almost all diclofenac was encapsulated in SLNs. After storage of nanoparticles at 2–8°C, size of nanoparticles was increased, but zeta potential remained unchanged. Loading efficiency of drug in prepared SLNs was almost unchanged after two months of storage.

Variation of drug concentration in vitreous and aqueous humor samples after intravitreal injection of SLNs containing diclofenac was summarized in Table 2. Drug concentration in eyes injected with SLNs was significantly higher than left eyes injected with commercial product up to 4 hours after intravitreal injection (*p* < 0.05). Diclofenac was quantified in samples up to 48 hours after intraocular injection. Four hours after intravitreal injection, concentration of diclofenac in vitreous and aqueous humor of eyes that received SLNs was, respectively, 2.5 and 6.5 times higher than the eyes injected with commercial form of drug.

## 4. Discussion

In the present study, SLNs as carrier of diclofenac have been prepared and characterized. SLNs were used as carrier of this drug due to some advantages such as maintaining drug concentration in therapeutic range for a long time, reduction of ocular toxicity, and slow and controlled drug release. In order to increase the drug residence time in vitreous, application of colloidal carrier system has been evaluated in previous studies [13–15]. Different drug carriers such as liposomes [16], microspheres [17], thermogel [18], and insert [19] have been used. For example, Abrishami and his colleagues [16] have evaluated* in vitro* and* in vivo* characteristics of bevacizumab nanoliposomes. After the intraocular injection of liposomal formulation, vitreal concentration of drug was higher compared to the injection of commercial form in different times even 42 days after injection. Nanoliposomal formulation provided more stable concentration for a longer time.

SLNs have been used as a suitable carrier for encapsulation of hydrophobic drugs, but most of hydrophilic drugs had low encapsulation efficiency due to hydrophobic nature of SLNs matrix [20]. Encapsulation efficiency of nanoformulations is affected by different factors including preparation method, nature of drug, and other formulation ingredients. To solve the problem of poor encapsulation of drugs in SLNs, different strategies have been used to improve the liposolubility of drugs. In the present study, we used diclofenac base instead of diclofenac sodium. Almost all of feed diclofenac was loaded into SLNs matrix.

In the study of Barañano et al. [8], pharmacokinetic evaluation of diclofenac sodium commercial formulation showed the rapid clearance of this drug after intravitreal injection. No drug was detected 24 and 48 hours after intraocular injection of 0.3 mg diclofenac sodium. But, in the present study, concentration of diclofenac sodium after intraocular injection of both commercial and SLN formulation was determined in vitreous and aqueous humor samples until 48 hours after intraocular administration. This difference can be related to the sensitivity of analysis method.

On the other hand, differences observed between SLN and commercial formulation showed that SLN could be a choice of slow-release formulation for ocular drug delivery. This kind of formulation provided longer appropriate therapeutic concentration in eye. In addition, drug concentration in vitreous and aqueous humor was evaluated in the present study, but the concentration in other parts of eye (e.g., cornea, iris, ciliary body, conjunctiva, sclera, and retina) was not determined. So, the reported concentration may be lower than the real concentration in eye. It has been shown that basic and lipophilic drugs could attach to melanin [21]. This attachment significantly reduces pharmacological activity of drugs [22]. So, melanin in the iris and ciliary body may change drug concentration in posterior [23] and inferior parts of eyes [24]. Determination of drug concentration in the other parts of eye is suggested for future studies.

## 5. Conclusion

Results showed that SLN formulation could provide higher vitreous and aqueous humor concentration for a longer time in rabbit eye compared to commercial formulation. SLN formulation can increase intraocular injection intervals and decrease the drug toxicity due to multiple drug administration.

## Figures and Tables

**Table 1:** Mean size, polydispersity index (PDI), zeta potential, and encapsulation efficiency of solid lipid nanoparticles containing diclofenac after preparation and two-month storage at 2–8°C (mMean ± SD, *n* = 3).

	After preparation	After one month	After two months
Mean size (nm)	172.7 ± 6.3	216.4 ± 10.2	269.5 ± 2.8
PDI	0.254 ± 0.001	0.184 ± 0.044	0.150 ± 0.083
Zeta potential (mV)	−0.9 ± 2.0	−1.5 ± 0.5	0.5 ± 1.3
Encapsulation efficiency (%)	95.1 ± 4.2	98.2 ± 0.8	98.0 ± 2.4

**Table 2:** Concentration of diclofenac (*µ*g/mL) in vitreous and aqueous humor samples after intravitreal injection of solid lipid nanoparticles containing diclofenac (mean ± SD, *n* = 3). Right eyes and left eyes were injected with solid lipid nanoparticles containing diclofenac and diclofenac commercial injection, respectively (dose = 0.3 mg).

Time (hour)	Vitreous samples		Aqueous humor samples	
	Right eyes	Left eyes	Right eyes	Left eyes
1	88.94 ± 13.03	33.47 ± 22.45	10.220 ± 4.943	1.564 ± 0.547
4	58.93 ± 17.86	3.87 ± 2.11	8.661 ± 3.419	1.988 ± 1.469
12	1.94 ± 1.31	0.30 ± 0.22	0.200 ± 0.102	0.181 ± 0.109
24	0.24 ± 0.14	0.12 ± 0.07	0.133 ± 0.041	0.113 ± 0.084
48	0.14 ± 0.01	0.09 ± 0.06	0.076 ± 0.023	0.008 ± 0.007
